# How public health crises expose systemic, day-to-day health inequalities in low- and-middle income countries: an example from East Africa

**DOI:** 10.1186/s13756-022-01071-5

**Published:** 2022-02-14

**Authors:** Alicia Davis, Tiziana Lembo, Emma Laurie, Edna Mutua, Kathrin Loosli, Mary Nthambi, Amy Nimegeer, Kunda Mnzava, Elizabeth F. Msoka, Fortunata Nasuwa, Matayo Melubo, Gabriel Shirima, Louise Matthews, Shona Hilton, Stephen E. Mshana, Blandina T. Mmbaga

**Affiliations:** 1 School of Social and Political Sciences/Institute of Health and Wellbeing, University of Glasgow, 27 Bute Gardens-Rm 221, Glasgow, G12 8RS UK; 2 The Boyd Orr Centre for Population & Ecosystem Health, Institute of Biodiversity, Animal Health & Comparative Medicine, College of Medical, Veterinary & Life Sciences, University of Glasgow, Glasgow, UK; 3 School of Geographical and Earth Sciences, University of Glasgow, Glasgow, UK; 4 Medical Research Council/Chief Scientist Office (MRC/CSO) Social and Public Health Sciences Unit, University of Glasgow, Glasgow, UK; 5 Department of Microbiology and Immunology, Catholic University of Health and Allied Sciences/Bugando Medical Centre, Mwanza, Tanzania; 6 Kilimanjaro Clinical Research Institute, Kilimanjaro Christian Medical Centre, Moshi, Tanzania; 7 Nelson Mandela African Institution of Science and Technology, Arusha, Tanzania; 8 Kilimanjaro Christian Medical University College, Moshi, Tanzania

**Keywords:** Health inequities, Antimicrobial resistance, Qualitative data, East Africa, One Health

## Abstract

**Background:**

The current Coronavirus disease pandemic reveals political and structural inequities of the world’s poorest people who have little or no access to health care and yet the largest burdens of poor health. This is in parallel to a more persistent but silent global health crisis, antimicrobial resistance (AMR). We explore the fundamental challenges of health care in humans and animals in relation to AMR in Tanzania.

**Methods:**

We conducted 57 individual interviews and focus groups with providers and patients in high, middle and lower tier health care facilities and communities across three regions of Tanzania between April 2019 and February 2020. We covered topics from health infrastructure and prescribing practices to health communication and patient experiences.

**Results:**

Three interconnected themes emerged about systemic issues impacting health. First, there are challenges around infrastructure and availability of vital resources such as healthcare staff and supplies. Second, health outcomes are predicated on patient and provider access to services as well as social determinants of health. Third, health communication is critical in defining trusted sources of information, and narratives of blame emerge around health outcomes with the onus of responsibility for action falling on individuals.

**Conclusion:**

Entanglements between infrastructure, access and communication exist while constraints in the health system lead to poor health outcomes even in ‘normal’ circumstances. These are likely to be relevant across the globe and highly topical for addressing pressing global health challenges. Redressing structural health inequities can better equip countries and their citizens to not only face pandemics but also day-to-day health challenges.

## Background

Some of the greatest global health crises continue to expose persistent and systemic health inequalities, demonstrating that universal health care access is still beyond the reach of many, particularly the world’s most vulnerable populations [1]. The Coronavirus disease (COVID-19) pandemic is no exception [2]. Whilst ostensibly all populations are at risk, it is those who are most exposed to uneven distribution of resources or access to health care that will likely pay the heaviest price. The pandemic risks worsening long-standing structural fault lines within and between countries, exacerbating existing health disparities and the unequal burden faced by the poorest in our global society thus exposing structural violences pervasive across the globe [3, 4]. The attention afforded to COVID-19 presents a unique opportunity to both learn from research on existing global health challenges and galvanise political momentum to create more equitable responses to such challenges or for day-to-day health care.

COVID-19 demonstrates how world health officials’ advice and guidance to the general public, health professionals and governments may be out of reach for many or may even be disregarded [5]. Handwashing is a challenge for those with poor sanitation provisions, and procurement of basic goods for personal protection is problematic when markets distort in response to global demands. Social distancing can be unattainable for those living in close quarters or crowded conditions or can contravene social norms. Similar to the COVID-19 emergency, other health crises, e.g. Human Immunodeficiency Virus Infection [6, 7], Influenza A virus H1N1 [8], Ebola [9], and Zika [10], have all suffered from the same imbalances and continue to reveal the challenges of global equity in preparation and response. This paper will focus on antimicrobial resistance (AMR) as a striking example of these disparities and global health inequities. The issues faced in tackling AMR, we argue, parallel with many of the issues in responses to COVID-19 and other health crises. Not only can lessons be learnt from existing research on AMR to overcome COVID-19, COVID-19 could also be an opportunity for more equitable responses to other major global health challenges.

AMR is a growing concern globally, with up to 10 million deaths estimated by 2050 and major costs to health-care systems [11]. While COVID-19 has galvanised significant attention worldwide, AMR is a slower, quieter, often ‘invisible’, yet much more persistent threat. The most substantial impacts of AMR are felt in low-and middle-income countries (LMICs), in populations who hold the largest burdens of poor health globally [12, 13]. In these areas, access to high-quality drugs is essential to preserve the health of humans and animals given the considerable impacts of infectious diseases. In LMICs, animals are critical to life and livelihoods. For example, in Africa 70% of the population depends on livestock [14]. Inadequacies in human and veterinary health infrastructures [15, 16] can contribute to the development of resistance for a variety of reasons, such as poor access to diagnostics, professional advice, or even basic sanitation and hygiene [17, 18]. Where care options are limited, health providers, patients, and farmers use antimicrobials in lieu of other measures [18]. Understanding the constraints patients, farmers and health practitioners face in particular environments and the local contexts in which they occur [19] is critical for tackling global health crises and devising tailored solutions. As such, we will focus on rural communities in Tanzania to provide specific context.

Key to optimal health care provision is supporting health systems broadly [18]. The health system in Tanzania has a complex history that transitioned from socialist public health care to a neoliberal model inclusive of user fees and for-profit health facilities [20]. Today the health system is a multi-tiered combination of public, private-not-for-profit, and private-for-profit health care which is used in conjunction with ‘traditional healers’ [21] or self treatment with herbal remedies. In this paper, we highlight areas within the Tanzanian health system that exemplify how global health inequities play out within the context of AMR. We present data from our ongoing research on AMR in relation to human and veterinary health to demonstrate the challenges faced by health care workers, patients and farmers alike at multiple levels and scales. We conclude by drawing together our empirical examples with a broader discussion about the implications for global health inequities.

## Methods

### Data collection

#### Sites and participants

The human and animal health systems in Tanzania exist as multi-tiered structures with zonal and regional facilities placed at the highest tier, district provisions in a middle tier and village provisions at the lowest tier. Data were collected within communities (April–May 2019) and at higher tiers of the health system (April 2019–February 2020) (see Table 1). Within communities, 14 focus group discussions (FGDs) with 121 participants and two in-depth interviews (IDIs) were held in three villages in three regions (Kilimanjaro, Arusha and Mwanza) of northern Tanzania representative of key livelihood strategies predominant in rural East Africa (agro-pastoral, pastoral and rural smallholder). Villages were matched based on human/livestock population sizes, number of sub-villages and access to healthcare in the form of hospitals/dispensaries, veterinary offices and drug shops. Two topics, animal or human health, were addressed in separate FGDs, each discussed with either the respective healthcare providers (providers) or members of the community. Within higher tiers, a total of 39 IDIs and 2 FGDs (totalling 15 participants) targeted providers (ranging from nurses to consultants) in five health facilities in Kilimanjaro and Mwanza regions. See Table 1 for overview of sites and participants.

**Table 1:** Number of focus group discussions (FGDs) and in-depth interviews (IDIs) conducted in three regions of northern Tanzania to discuss human and/or animal health

Stake-holder type*	Topic discussed	Stakeholders present	Tier	Number of FGDs/IDIs**	Locations (Village’s Region)	No. participants/village
AHP	Animal health	Animal healthcare providers	Lower	3	Kilimanjaro	1 (IDI)
					Arusha	6
					Mwanza	1 (IDI)
CAH	Animal health	Community members	Lower	3	Kilimanjaro	10
					Arusha	12
					Mwanza	8
CHH	Human health	Community members	Lower	3	Kilimanjaro	6
					Arusha	13
					Mwanza	7
HHP	Human health	Human healthcare providers	Lower	3	Kilimanjaro	5
					Arusha	8
					Mwanza	11
HHP	Human health	Human healthcare providers	Higher/middle	39 IDI/2 FGD	Kilimanjaro	38 (30 IDI + 8 in FGD)
					Mwanza	16 (9 IDI + 7 in FGD)
HC	Human and animal health	Community members	Lower	4	Kilimanjaro	13
					Arusha	9
					Mwanza	13
Total				57 interviews		136 participants

*The following stakeholders were involved: animal healthcare providers (AHPs), community animal health (CAH) workers, community human health (CHH) workers, human healthcare providers (HHPs), and community members. In the health campaign (HC) FGDs, animal health and human health were discussed with community members

**All but 2 interviews in communities were in FGD format, but IDIs were conducted when there were no other participants/providers available. All interviews in higher tiers were IDIs

#### Interview protocols

Given that data collection was divided between community level and higher tiers of the health system, and between human and animal health, and providers and community members, there were variations in interview schedules that centred around particular groups and their particular experiences and knowledge. However, there were distinctive overlaps in questions, wording, tone, and approach. Each interview schedule included questions about AMR knowledge and attitudes, awareness of public health campaigns and messages, communication with patients/providers, and diagnostic and treatment processes, including self-treatment. Additional questions were asked of community members about access to services and local infrastructure, trusted sources of health information, priority health issues of concern (human and/or veterinary) and specific self-treatment options. Community level protocols also investigated general meanings of health, wellbeing, and the qualities of good health campaigns. Higher tier providers were asked about IPC measures, prescribing practices, and personal motivations for working in the health system.

All interviews in both data collection streams were conducted or moderated by Tanzanian research assistants fluent in the main languages spoken in the study locations, Swahili or Maasai. Interview protocols were developed to steer the discussion but retained a degree of flexibility in order to capture the natural flow of the conversation. Notes were taken throughout the FGDs by dedicated note takers and the conversations were also audio recorded. All data were collected following strict ethical protocols and approvals via the University of Glasgow, the National Institute for Medical Research in Tanzania (NIMR) and with verbal and/or written consent from all participants. Details of our ethical protocols can be found in an ethics statement in the acknowledgements.

### Data analysis

Interviews for both streams were recorded (when consented to), transcribed and translated to the English language by Swahili and Maasai speakers fluent in English. Where interviews were not recorded (n = 5), analysis was conducted directly from fieldnotes.

The interviews were analysed using NVivo 12 (QSR). A coding matrix was built from a mix of inductive codes (deriving from the data themselves) and deductive codes (informed by research questions and literature) and applied across the data sets. An association matrix between individual codes was used to group them into broader themes. Further review of literature contextualised these emergent themes.

### Role of the funding source

This project was funded by the Antimicrobial Resistance Cross-Council Initiative through a grant from the Medical Research Council, a Council of UK Research and Innovation, and the National Institute for Health Research, project number (*MRC/AMR/MR/S004815/1*). The funders had no involvement in the study design, data collection, analysis, or interpretation of the data nor in the writing of or decision to submit this paper.

### Patient and public involvement

This project was coordinated in consultation with Tanzanian health authorities including the Ministry of Health, Community Development, Elderly, and Children and their National AMR Coordinating Committee. Community-based work was developed with District-level medical and veterinary officers who participated in a workshop to identify key areas of research need within their districts as it relates to AMR, drug procurement and use, and health care access. We designed our research instruments with the aim to address these specific needs. The broader project upon which this study is based directly involves communities, and particularly local leaders and health providers, to co-create locally responsive health interventions. Study participants have so far included key stakeholders such as health seekers and their health providers in order to ascertain representative experiences and voices. Result dissemination is an integral component of our research program and results will be presented to communities, district officials, and the Ministry in order to devise relevant solutions.

### Limitations of the research

There were limitations in this study. The first is that it was part of a ‘preliminary’ data collection period of a broader study, therefore a full representation of types of health providers and community members is limited. However, through our sampling protocol in this initial data collection period, we attempted to be inclusive of as many providers as possible and we included key, respected community members and leaders. Our community data was collected from three communities with similar access to health facilities and programs, however communities with greater/lesser access were not represented. This data is also solely of a qualitative nature, which may be limiting to some readers and in representing a broader scope of practice and experience around AMR. However, in our main data collection period, we have attempted to address these limitations. For example, we have included a broader range of providers at higher tier hospitals and pharmacies and we have conducted a wider array of mixed methods with a larger number of participants. We are currently collecting household surveys, health provider exit surveys, in-depth interviews with patients and providers across communities and facilities in a range of locations. This will provide additional information and understanding of the themes discussed below.

## Results

Multiple overlapping themes emerged that directly relate to both individual health care experiences and systemic health challenges. We organise these themes according to three broader issues relating to health: *infrastructures, access and choice, and health communications*. First, there are challenges around infrastructure and availability of vital resources such as healthcare staff and supplies as well as broader infrastructures such as roads, clean water, and communications. Second, health outcomes are predicated on patient and provider access to services as well as social determinants of health including economic constraints, one’s sense of agency, and self-treatment processes. Third, health communication is critical in defining trusted sources of information, and narratives of blame emerge around health outcomes with the onus of responsibility for action falling on individuals. The nuances of health experiences, beliefs, behaviours and meanings for patients and providers are elaborated upon through providing direct statements from our interviewees.

### (1) Infrastructures

We define infrastructures of health as they relate to the numerous aspects of health systems and their broader support structures. Our participants discussed both the challenges and advantages they face regarding the infrastructure and concomitant supplies required for a functioning health system. These span from personal protection, including procedures for infection, prevention and control (IPC) and access to personal protective equipment (PPE), to laboratory support for diagnostics. We consider staff (and their time) as well as availability and quality of drugs as key entities of health infrastructure. The broader support structures include components linking people to a health system or health information, such as transportation, roads and basic communication networks (phones/internet). Specific examples from across the health system (i.e. middle/higher tier hospitals and community settings) are detailed below.

#### Understaffing and lack of supplies

Our findings indicate that limited health infrastructure (IPC, PPE, staffing, diagnostics) poses challenges to providers and patients across the multiple tiers of the health system, albeit it to varying degrees.

Providers reported shortages of doctors, animal health providers, nurses, clinical officers and laboratory technologists as important issues in their facilities and locales. This resulted in heavy workloads and time pressures when serving patients. Both human and animal health providers acknowledged that the serious shortage of professionals generates waiting times that discourage initial use or returns to facilities by patients and farmers (quotes (Qs) 1 and 2):

*Q1. The clinic today has few doctors and that is what needs to be looked into because like until now there are patients who are still waiting for service because of the shortage of doctors. They need to increase doctors and nurses, if possible, because the clinic is supposed to end even at 2:30pm but I will be here until 5:30pm. Don’t you see that is tiring? But what can we do?—*Health Provider, higher tier

*Q2. … due to the shortage of experts, for example here in the whole ward [an administrative unit smaller than a district] we have only one known [livestock] expert and the ward is big and the livestock are many. For example, we have 31,900 cattle according to the recent count [a census count in 2018]... Therefore, treating all these animals is difficult, even if you are told to treat all these animals you can’t do it because there are too many.—*Animal Healthcare Provider, lower tier

Livestock owners often do not seek professional care because the relevant government livestock field officers (LFOs) are not available, live far away or charge too much to travel long distances to their farms. This leads to their reliance on lay experts, such as retired veterinarians or other paraprofessionals (themselves rarely available), who become the best solution until the LFO has time to come to households personally.

Water shortages or lack of hand washing stations, soap and gloves across health facilities (especially lower tiers) compromise provider’s abilities to adequately implement IPC measures (Q3).

*Q3. There is a time when we don't even have gloves, brother, water is an issue. The soap itself today we have comes from having an argument, you find that things are not there. What do you do and the patient is there, will you say you can't serve a patient because you have no gloves? No. The other time the gloves are the big challenge, the gloves are not available, and the patient is there what will you do?—*Health Provider, Outpatient Department, higher tier

Even when facilities have adequate supplies, further issues constrain implementation of IPC, particularly of concern to providers at higher tier facilities. They stated practices were hampered by staff attitudes, workloads and even building design (i.e. where sinks or hand cleaning supplies are placed) (Q4).

*Q4. The challenges* [in IPC], *first I can say is the habit of the health practitioners. Sometimes we, if for instance, the issue of hand washing and scrubbing, [the] majority they do not want to follow the steps, it means we do act based on our personal habits so that one is a challenge – it means practicing based on experience rather than the guidelines of IPC.—*Health Provider, Medical Department, higher tier

There is variation in the availability of laboratories, testing equipment and diagnostic supplies across the health system. Larger, higher tier facilities tend to be better equipped and have more diagnostic capabilities. Though uncommon, some of these facilities have problems with keeping stocks of supplies consistently and equipment maintained. Lower tier facilities often have to refer patients to higher tier facilities because of complete lack of testing or diagnostics, but this usually leads to higher costs and stress for patients (most of whom are poor). Even when diagnostics are available, other factors, such as lack of training can prevent their use (Qs5–8).

*Q5. Here [in this health facility] we use normal tests which use the microscope only. We do not do culture tests.—*Human Health Provider, higher tier

*Q6. We have no test for Brucellosis to prove that Brucellosis is a problem here.—*Human Health Provider, lower tier

*Q7. …you know sometimes you might find our biochemistry machine is not working today so we have to send them [patient samples] back, but if everything is fine they can be done here. … So here I have heard if the biochemistry for full blood picture is not working we send them to X (zonal hospital). For CT scans they have to go outside to X Region or some other place.—*Human Health Provider, higher tier

*Q8. Because we don’t have diagnostics to do cultures or whatnot, but big hospitals they test even for the bacteria which causes pneumonia. We are just seeing that a person is not breathing well.—*Human Health Provider, lower tier

Similarly, medicine availability varies both across the health system tiers and geographically, with wide regional heterogeneities. Providers highlighted lengthy processes for procuring drugs through government channels, which force them to send patients to obtain medicines elsewhere, particularly pharmacies (Qs9–11).

*Q9. There are a lot of challenges [with the Medical Stores Department] because they are working with the government so you may find maybe we are purchasing an order for a drug like it takes three months, six months for it to come. So instead we send patients to go get them from outside pharmacies.—*Health Provider, Cancer Department, higher tier

*Q10. [we get drugs] at the pharmacy, because if you go to the hospital you are told there are no drugs. We go get [them] from the pharmacy.—*Community Members

*Q11. Here in our village, they cannot have those medicines because they do not have diagnostic tools to test diabetes, and if you put those medicine here, they will expire.—*Community Members

Broader issues arose about the lack of supporting infrastructure (roads, communication networks, water) that enables individuals to access health care. This is often experienced acutely in rural villages (Qs12–13).

*Q12. Respondent (R): Now sir (elder), what percentage of old people living here will even open Whatsapp? Interviewer: Hmm, does it work? R: …and also the network coverage here is not very good.—*Community Member

*Q13. R4: For example there are people here who have phones but they just use them to listen to music. If you send a text message, R1: …they might not read it.—*Community Members

### (2) Access and Choice

Without appropriate infrastructure, patients’ and providers’ access to care is limited. Thus, we define access and choice as encompassing the availability of, transportation to, cost of, and trust in health care as well as the agency to make and act on health decisions. Access also includes access to basic sanitation and needs (nutrition/food/ water) that allow people to stay healthy. We not only consider access to *what* is available, but also *who* can access the available infrastructure, as well as *when* and *why*. Access is directly related to the health decisions and choices people have, particularly within constrained and pluralistic health systems. Agency, the ability for people to be empowered to make choices, is affected by the information at hand to enable decisions, the availability of choices to make, the social and economic constraints that affect choices, and, in the specific realm of health care, the functionality of a health system. With these issues in mind, we describe several of these key factors including the economic constraints people face and issues of empowerment in health decision making. Self-treatment serves as a critical example of the interface between access and choice in this complex health landscape.

#### Economic constraints

Access is also compounded by individual and institutional economic precarity. Both patients and providers identified costs as the foremost issue affecting their access to health care and health decision making. Costs include not only direct payments like hospital fees or drug prices, but also indirect or non-monetary expenses such as transport costs or time invested to seek treatment. Farmers mentioned that the costs of seeking professional help is high because they not only have to pay for their animal’s treatment, but also the transport costs of the veterinarian to come to their homesteads, often over long distances (Q14).

*Q14. Because it is expensive for the livestock officer, the to and fro costs, that is why I am telling you, people ask for advice, and they go for self-treatment. Because otherwise the costs go up.—*Community Member

Health providers also noted where they are restricted by cost (Q15).

*Q15. Yes, they are available [resources to implement IPC] but they are not enough. Things like hand sterilizers are expensive so they are not available in each and every department as is supposed to be. Sometimes you have to keep them in the clinic and the in theatre in the ward…—*Health Provider, Medical Department, higher tier

Insurance costs were a recurring issue in all tiers. Although Tanzania is rolling out ‘affordable’ health insurance programmes, these have not reached many remote rural communities. Additionally, in the few areas where it is available (only one of three research villages) insurance is not only an expensive, often unaffordable, ‘upfront’ cost, but it can be of limited use if there are drug shortages or lack of facilities to visit, thus producing a double financial burden (Qs16 and 18). Finally, providers feel that when patients have insurance, they become more demanding for preferred medicines or treatments (Q17).

Economic constraints were often expressed this way:

*Q16. I have struggled, I sold my goat, I sold my chicken and paid for my health insurance, let me go there* [to the hospital] *for treatment. At the hospital, I was asked to show my health insurance card, the doctor took the insurance card and … they say… go to the pharmacy to buy medication because there is none available [here], so they need to be bought. So I ask myself, ‘now I paid 30,000/*= *(£10) for the insurance and the bill from the pharmacy for the medicines is 40,000/*= *(£15)’. Coming back home, a person would say to people, I don’t see the importance of this insurance card. I paid 30,000/*= *for insurance then I go there [the hospital] and pay 40,000/*= *for medication. Don’t you see that we will die?—*Community Member

*Q17. Those who are using the national health insurance scheme, they have a high chance of pressuring the doctor because they say ‘I am paying for my health insurance so prescribe these medications for me’, but those who are paying from their pocket do not have much pressure because sometimes if you prescribe for them very good medication and it is expensive, they cannot afford to buy it.—*Health Provider, higher tier

*Q18. Many people die because of this. You don’t have health insurance and you have no money to buy it.—*Community Member

#### Empowerment/agency

Empowerment to make good health choices (as a patient or provider) is predicated not just on individual choice, but the underlying constraints people face within which decisions are made. These health seeking decisions can be impacted by perceived severity of disease, understanding causes of disease, shared (dis)information, trust in expertise or personal networks/relationships, prior experience, and cultural norms and beliefs. For providers, social status related to age or position can prevent younger clinicians from feeling empowered to make decisions. Whereas for patients, personal health experiences including sharing health experiences with one’s family, neighbours and community can shape health seeking decisions (Q19).

*Q19. R1: …the first one to trust is myself, I must be sure of my own life and my health. After that I will go and see the doctor and he will confirm and tell me that I have this and this problem and we decide what to do...R2: I think the first trusted source is the patient himself, because if the patient goes to the hospital, there are other illnesses he can explain personally and the rest to be shown by the test results. I think it is that way, full stop.—*Community Members

In rural communities, facing challenges to accessing critical health infrastructure (clinics, providers, etc.), self-reliance has emerged as a critical strategy of care. While some people feel that self-treatment is their only choice, others express it is their preferred choice, but the reasons for this are multifaceted as we discuss below.

#### Self-treatment

Self-treatment is a complex issue with myriad causes, effects, and attitudes affecting it. Drivers of self-treatment are multiple, relating to preference, cost, agency and freedom, cultural norms, and availability or access of health provisions. Forms of self-treatment range from use of local herbs and home remedies (which have no cost) to over-the-counter medications (e.g. paracetamol and cold tablets), to antibiotics which relies on prior experience or advice from social networks or drug sellers. Provider attitudes about patient self-treatment can range from ‘understanding’ to ‘admonishment’ and these attitudes are often reflected in the provider-patient experience. Patient attitudes about self-treatment also span from perceived ‘lack of choice’ to a ‘preference for’ using it. Decisions are often made on the grounds that self-treatment is cheaper, easily accessible, broadly available and commonly preferred. When faced with illness, people self-assess the severity of their condition and often choose a path based on this condition as well as their means. People also continually re-assess their status which may alter the path of action (Q20).

*Q20. Some neighbours go to the [traditional] healer, others when they wake up, if they feel dizzy, they just go into the woods and cut some roots, boil, then drink .... They boil roots mixed with leaves and they consider it to be a medicine. If it fails to cure, that’s when they go to the health centre.—*Community Members

People dually recognise the expertise of doctors but also acknowledge the importance of their own practices (Q21). The contrasting views emerge especially when people think self-treatment may be seen as ‘bad’ behaviour, as many health providers believe and emphasise.

*Q21. R6: Self-treatment is just a first aid that you can provide to yourself. You are not an expert so you can only treat yourself as first aid.*

*R11: Self-treatment among us is low because we are not experts.*

*R8: We cannot treat ourselves because we are not doctors.*

*R7: We do treat ourselves because we go to the bush slaughter a bull and drink soups made of herbs.*

*R12: Sometimes you might go to the hospital three to four times and if you do not recover, we take herbs, and that cleans the excessive bile.—*Community Members

### (3) Health Communication: communication, information and relationships

As important as it is to acknowledge both the choices people have and the constraints of the health system, it is also vital to understand health communication, which is itself impacted by infrastructural constraints and systemic inequities. We consider the following key elements of health communication: (1) the human networks and relationships that enable communication about health, (2) narratives about health and disease, and (3) how these narratives influence health seeking experiences.

#### Communication networks and relationships

Our research reveals that both patients and providers have access to formal and informal networks for obtaining health information. These include direct communication between providers and patients during the health seeking process as well as communication that occurs in formal health campaigns run by the government or non- governmental organisations (NGOs). Sources of information can vary from one’s self (through past experiences, within and outwith the health system) to others in one’s social network, from family, friends and community leaders to health experts. How one is defined as a health ‘expert’ is tied to perceived and actual levels of ‘expertise’ that reflect hierarchies of social status. Within communities, respondents named their leaders, teachers, local healthcare providers (human and animal health) and spiritual leaders as important sources for health information (Qs22–24).

*Q22. We share advice amongst ourselves. Someone might tell you, “I have injected my animals this way” and you also imitate that.—*Community Members

*Q23. I saw that the best people are the chairpersons and the village executive officers, we use them to spread information.—*Community Members

*Q24. Letters are dispersed and read in churches and in the mosque maybe many will see. People can go to the health center in X. This one [recent health campaign] was really successful and people participated.—*Community Members

Key components of communication frameworks are the human relationships that act as conduits for information flow. These two-way relationships are often uneven because of social status. However, they are a means to transfer information within the treatment process and which can facilitate successful health campaigns. Social norms and hierarchies dictate that those in positions of power or authority have more social capital and are thus considered to have more knowledge (Q25) and should be listened to.

*Q25. I trust the livestock officer because he has studied diseases.*—Community Member

*Q26. They [farmers] have built trust and share knowledge amongst themselves.—*Animal Healthcare Provider, lower tier

This establishes clear divisions between patients and providers. Thus, relationships at all levels can be imbued with trust or mistrust. Information acquired either from experience (Q26), formal training or through social capital were considered critical components of trust from the perspectives of patients, farmers and providers (Qs27–28).

*Q27. You go to the doctor, the doctor will just kind of look at you, you tell him that you are having chest ache, coughing, they just look at you and then they write a prescription, and tells you “go to the dispensary window”. Ok, now I fail to trust them a bit, they were supposed to advise me, to get a diagnosis. Okay so we fail to know what we are suffering from. I have just said that I am suffering from cough, they were supposed to tell me to go and get tested (by spitting my phlegm) and the expert would test it.—*Community Member

*Q28. Of course, there are various factors that contribute to the receipt of information, there are those patients who are educated [they] will understand you first, when you give them information. Uneducated [people] take time. You have to tell her two, three times the same thing. There are others who, according to their culture, they don't value women, so you as a woman [doctor] can’t really tell them anything. So you have to use someone else, so it depends on the type of patient you have.—*Resident Pediatric Doctor, higher tier

This is also true amongst providers themselves (depending on where in the health system someone is) (Q29). Clinicians described hierarchies of trust within their own networks. Communications from senior male doctors are considered more believable and authoritative by patients. In contrast, more junior doctors (interns), female doctors and nurses were more likely to be challenged or disbelieved by patients.

*Q29. We need to start educating people, but before we educate people we need to educate each other.—*Resident Pediatric Doctor, higher tier

#### Narratives of health

In the short amount of time providers have with their patients (Q1), when relationships are formed and levels of trust developed, narratives of treatment and of the health landscape more broadly are reinforced. The tone, discourse, and intent within these communication dynamics can reveal latent expectations for treatment or care options (of both parties), while also consigning responsibility over care. In addition, patient-provider communications are predicated on unshared understandings about biomedical theories of health and the body. Several distinct narratives arose from patients and providers based on the language used, the tone, and time providers spend with patients. These narratives place blame on the opposite party as holding the responsibility over care, as several quotes throughout the paper demonstrate. Additionally, narratives from providers about what people can and should understand about the biomedical process become set in this process. Narratives thus demonstrate tensions and sometimes strained undercurrents, particularly when services and infrastructure are wanting (Q30).

*Q30. …you will see some patients they will come and insist that they need antibiotics and some of them, I don’t know they have the mentality, they believe that injections are more effective than tablets so they will request an injectable antibiotic, which is not right.—*Medical Doctor, Specialist, higher tier

Expectations of responsibility can put pressure on the providers to prescribe drugs like antibiotics or painkillers which the patient is familiar with, instead of conducting a thorough analysis to diagnose the disease. Patients may also seek ‘treatment’ at a pharmacy to buy known drugs previously prescribed for the same symptoms instead of going back to the facility (Q31).

*Q31. They’re not coming to seek advice, they [a patient] just come and say please give me Panadol, if you try to ask what they are suffering from, they will start asking you, “eeeh, hey now, why are you asking all those questions?—*Human Healthcare Providers, lower tier

These narratives also fit with broader discourses about responsibility over health care and blame for antimicrobial “mis” use, which is often assigned to the individual “patient” (or “user” or “consumer”). Thus, when doctors talk about AMR, it is often consigned as the patient’s fault (Q32).

*Q32. We see many patients when you prescribe antibiotics like for five, or seven days they will take it for three days and they come back with fever. You ask them, “did you finish that dose?” They will say no. So, they don’t understand the magnitude of not completing the course of antibiotics so I think they still are not aware about it.—*Medical Doctor, Specialist, higher tier

#### Impacts of communication on health

As discussed above, communication and relationships between providers and patients have an impact on perceived quality of care. Patients report that information is not clearly communicated to them, including diagnoses, which they consider poor service and dismissiveness by providers. They feel their needs or expectations as patients are being neglected or ignored, which impacts people’s trust in a health facility and in the system, causing some to decide not to return to seek care (Q33).

*Q33. Poor service from the doctors [was her stated concern]. For example, two days ago my baby was sick, I took the baby to the [government] dispensary and the baby was given medicine given to people with asthma. First off, I met the doctor who said, ‘ehee?’ I explained my child’s situation, that the baby is suffering from chest ache and flu. Without even listening [to me] nor even asking me when the situation started or whether the baby had fever or not …[he] ordered me to go to the dispensing window, I was given piriton, paracetamol and medicine like those given to asthmatic patients. I will never go there again. If I have money, I will go to a private health center...—*Community Member

Despite all these issues, providers (and patients) recognise that improving communication between themselves and their patients is crucial, especially in the context of AMR (Q34).

*Q34. Interviewer: what do you consider the biggest challenge in communicating AMR to patients? Respondent: the language of use, that is, how you will put it in simple language for them to understand, for the first time to come to treatment and then you tell the things, how to use the language and how you establish your relationship with your patient. Well, yes, there is the fact that you and the doctor / doctor and the patient can never talk at all. i.e. you arrive treating him/her or you're leaving, so how do you establish that relationship, they call it “rapport” [sic] with your patient.—*Resident Doctor, higher tier

## Discussion

Our study demonstrates major limitations of the health care system as well as a critical need to support it in a resource-limited setting, which is representative of many LMICs. Structural barriers exist that both limit access to and choice of health care, as well as preventing adequate care by providers. These barriers range from understaffing and medical resource limitations to broader infrastructural issues and are consistent across the different tiers of the health system, although they are more acute in rural areas. The impact of these resource limitations as well as the economic constraints that people in subsistence-based conditions face limit the range of options they have in terms of accessing care, sometimes triggering problematic, but understandable paths of self-treatment. In a context where access to information by professionals is constrained, broader, social networks of communication and information sharing become critically important. Finally, we emphasise how the narratives around health influence patient–provider relationships, and the health seeking experiences and practices that flow from those.

Sociocultural factors, such as poverty, food security and cultural norms, as well as systemic structural issues in health care, agricultural and economic systems have been implicated as important drivers of AMR [22]. Understanding the structural and societal factors that perpetuate these processes as well as local experiences is critical to the design of health interventions [13, 15]. Yet this is rarely done *with* the broad range of relevant actors that might be affected by these issues [23].

Here we address these gaps. Consistent with prior studies in East Africa we show similar structural barriers, i.e. understaffing, drug shortages and insufficient or unsuitable infrastructure [21, 24, 25] which in the Tanzanian context can partly be explained through the health system’s historical trajectory. Constraints to service provision due to scarcity of health centres or poor road infrastructure add further challenges for people seeking care [24, 26, 27]. Major variations and disparities in access to safe water and sanitation still exist across LMICs and sub-Saharan Africa more specifically [28], and compromise health delivery and outcomes. These barriers were acknowledged by nearly all our participants in human and animal sectors alike.

Structural, economic and socio-cultural barriers limit people’s access to and choice of healthcare with profound impacts on health. These impacts are seen in the everyday experiences of both providers, caught in difficult circumstances with limited choices, and of patients, people trying to make the best choices they can, given severe limitations. In short, poverty and confounding systemic oppressions (like the narratives of blame and individualisation of health outcomes) have a profound impact on people’s everyday lives, including their health. AMR narratives, responsibility over use and even behavioural solutions to AMR are often suggested to be instigated through “individualised action” and supposed “choice” [29]. Global narratives about AMR often fall back on the rhetoric of “misuse” or “prudent use” [30, 31] despite decided lack of actual options for people on the ground [29].

For this reason, we follow other health scholars who are increasingly turning to Johan Galtung’s work on structural violence to better frame people’s experience of health [4, 6]. In his own definition of structural violence, Galtung noted that “if a person dies of tuberculosis in the eighteenth century it would be hard to conceive of this as violence since it might have been quite unavoidable, but if a person dies from it today, *despite all the medical resources in the world, then violence is present”* [32]. The definition of structural violence hinges on the avoidable nature of suffering and death, and the concept situates individual experience within much wider historic and contemporary political structures [3].

Structural violence is being increasingly drawn upon, for it politicises, rather than naturalises, health inequalities. That is to say, the barriers to health detailed above, have been built, reinforced and actively protected by global political structures that prioritise profit and privatisation of health. This approach privileges the lives of the wealthy and abandons the world’s poorest, and, at its worse, actively abuses the bodies of the poorest to protect the wealthy. This is an often unchallenged yet widespread abuse [33], further exposing the notion of *global* health to be a “misnomer” [34].

## Conclusion

Health crises, including AMR, cannot be tackled while barriers to effective health care for the global majority persist. We must ensure that more barriers are not erected to further restrict access to health care, be that through the punitive ‘policing’ of drug use or increasing their price. Denyer et al. [35] suggest that antibiotics themselves are “infrastructure” often used as a “quick fix” to fill in infrastructural gaps. This further problematises AMR in terms of “individual” action and reveals the depth of systemic challenges related to AMR and health systems. As one doctor working at a regional hospital contended, in the future of AMR “there will be a lot of deaths because not all people will be able to afford the expensive drugs”. Our work in Tanzania has highlighted some of the day-to-day challenges faced by patients and providers. Like for COVID-19, this demonstrates why we have a collective responsibility to ensure that the fight against AMR is done in a way that tackles, rather than reproduces, existing health inequalities.

To do so, more voices need to be heard, yet global discussions on AMR have largely excluded the voices of African nations [36]. The situated experiences of patients and providers need to be listened to if this ‘view from below’ is to be understood [3]. We also need to push back against the global trend towards **‘**individualisation**’** of health which AMR ‘prevention and control’ is often couched in [37]. If the impetus of good health and staying healthy is put on individual action and choice, it fails to reflect the limitations, barriers, and poor structures of health that both patients and providers operate within. Thus, the success of future actions is dependent upon the availability of critical infrastructure, unobstructed health care access and clear communication strategies appropriate to the context.

## Data Availability

The datasets generated and/or analysed during the current study are not publicly available as the qualitative analysis is still ongoing. Identifying information may still be ascertainable from the raw interviews, but data summaries are available from the corresponding author on reasonable request.

## Abbreviations

AHP: Animal healthcare providers
AMR: Antimicrobial resistance
CAHW: Community animal health workers
CHHW: Community human health workers
FGD: Focus group discussions
HC: Health campaigns
HHP: Human healthcare providers
IDI: In-depth interviews
IPC: Infection, prevention and control
LFO: Livestock field officer
LMIC: Low-and middle-income countries
NGO: Non-governmental organisation
NIMR: National Institute for Medical Research in Tanzania
PPE: Personal protective equipment
Q: Quotation
